# Regional surnames and genetic structure in Great Britain

**DOI:** 10.1111/tran.12131

**Published:** 2016-07-07

**Authors:** Jens Kandt, James A Cheshire, Paul A Longley

**Affiliations:** ^1^ Department of GeographyUniversity College LondonLondonWC1E 6BT

**Keywords:** Great Britain, population genetics, cluster analysis, regional geography, surnames, geodemographics

## Abstract

Following the increasing availability of DNA‐sequenced data, the genetic structure of populations can now be inferred and studied in unprecedented detail. Across social science, this innovation is shaping new bio‐social research agendas, attracting substantial investment in the collection of genetic, biological and social data for large population samples. Yet genetic samples are special because the precise populations that they represent are uncertain and ill‐defined. Unlike most social surveys, a genetic sample's representativeness of the population cannot be established by conventional procedures of statistical inference, and the implications for population‐wide generalisations about bio‐social phenomena are little understood. In this paper, we seek to address these problems by linking surname data to a censored and geographically uneven sample of DNA scans, collected for the People of the British Isles study. Based on a combination of global and local spatial correspondence measures, we identify eight regions in Great Britain that are most likely to represent the geography of genetic structure of Great Britain's long‐settled population. We discuss the implications of this regionalisation for bio‐social investigations. We conclude that, as the often highly selective collection of DNA and biomarkers becomes a more common practice, geography is crucial to understanding variation in genetic information within diverse populations.

## Introduction

Recent social scientific interest in bio‐social relations – the ways in which individual biology interacts with social environments to produce specific patterning of health and social outcomes – have spurred more efforts to take into account biological information about individuals, including their DNA. Increasing data availability and continuous advances in computational power have made feasible the analysis of human DNA for a variety of research problems, and substantial investments are being made in the UK to assemble DNA records into purpose‐built genetic databases (Medical Research Council 2010; WTCCC 2014) or even as part of social surveys (McFall *et al*. 2012).

While today's genetic research is vaunted as contributing widely to social science research agendas, geographic variations in biological population characteristics have as yet received rather little attention. This contrasts with the history of genetics, which has been intimately bound up with the geography of populations and their movements. Traditional approaches to studying human genes (e.g. Cavalli‐Sforza *et al*. 1994; Cavalli‐Sforza 2002) clearly demonstrate that observing gene frequency in geographic space improves if not enables inquiry into the genealogy of populations. But while population genetics has been researched extensively at broader (global, continental) scales, identifying more granular, genetic structure within populations has proven difficult because of data and computational limitations (Biswas *et al*. 2009; Leslie *et al*. 2015; Winney *et al*. 2012).

Geneticists use the term ‘population structure’ to denote genetic differences between population sub‐groups, such as regions within a country – and it is such scales that likely provide a range of factors influencing individual bio‐social and biomedical outcomes (e.g. genome‐wide association studies: Bodmer and Bonilla 2008; Cardon and Bell 2001; WTCCC 2007). Fine‐grained population structure results from genetic differentiation within populations arising from a number of genetic and demographic processes (Cavalli‐Sforza and Bodmer 1971). In the simplest case, if population structure is not taken into account, the identification of causal pathways linking traits, genes, social and geographical factors may be confounded (Marchini *et al*. 2004). A promising and widely applied way of accounting for population structure is through geographic analysis of bio‐genetic or related population attributes (Cheshire 2014), but such studies have hitherto been based on incomplete or otherwise limiting data.

### Current studies on geographic population structure

Current studies of geographically detailed genetic structure can be divided into two types: those that use biological characteristics of sampled volunteers (e.g. Capelli *et al*. 2003; Cavalli‐Sforza *et al*. 1994; O'Dushlaine *et al*. 2010; Quintana‐Murci *et al*. 2004; WTCCC 2007) and those that use surnames as non‐biological surrogate indicators of population characteristics (Darlu *et al*. 2012; Degioanni *et al*. 2003; Jobling 2001). Scientists have recognised ever since the 19th century that many surnames are associated with distinctive population characteristics, and their association with genetic variation developed in the late 20th century (Lasker 1985, 6). Some correspondence between regional gene frequencies and local surname mixes has been verified in a variety of national contexts (e.g. Barrai *et al*. 2001, 2002; Boattini *et al*. 2012; Cheshire *et al*. 2013; Dipierri *et al*. 2011; Herrera Paz *et al*. 2014; Longley *et al*. 2011; Rodríguez‐Larralde *et al*. 1998, 2000). In the UK, many surnames emerged under locally or regionally varying naming conventions, for example by lineage, place names or regional description of occupation (Cheshire *et al*. 2009). This remains manifest in regional clusters of surnames in Great Britain today: in other words, most surnames do not occur evenly across the country, but rather remain concentrated locally, much like genetic variants (Cheshire and Longley 2012). Such concentrations persist over time, despite the relatively minor cumulative effects of internal migration.

The two types of studies have different strengths and weaknesses. Biological studies are close to the subject, as they collect either direct information on DNA structure or closely associated biological data (mtDNA, blood groups, antigens). Although no longer prohibitively expensive for bio‐social investigations, cost issues nevertheless restrict the size of attainable samples, and participation in investigations may systematically exclude some groups (e.g. religious groups, social classes, ethnic groups). Surname‐based studies are based on an indirect indicator of genetic structure, although they rest on specific assumptions about migration and may be subject to other confounding factors. Yet these studies are able to draw on nearly complete population registers, making it possible therefore to identify the geographical extent of sub‐populations within a population‐wide representation.

### Combining gene and surname geographies

In this paper, we seek to combine the strengths of both types of studies and relate a collection of DNA samples to Great Britain's regional surname geography. We argue that as biological concerns become part of social science research agendas, so hitherto neglected aspects of geographic sample designs need to be addressed in order to enable valid inferences about the population at large in any specific time period. Social surveys often seek to establish a basis to generalisation through prior stratification or posterior sample weights using indicators of demographic and social similarity that are deemed material to the survey's objectives. In biological and bio‐social research, however, it remains an open question as to what should be regarded as a representative biological sample that accommodates the effects of population structure. This question does not, of course, bear on the general problem that defining populations according to national boundaries or other geographical delimitations creates uncertainties with respect to non‐territorial shared population characteristics, such as genetics, identity or social attitudes (Nash 2013). This problem, although common to all sample‐based investigations in social science, becomes particularly apparent in the study of bio‐genetic population characteristics and points towards a tension between inductive and deductive approaches to defining populations. Although this paper cannot resolve this debate, it may nevertheless provide a first step by addressing the question of sample design in highly selective and unstructured DNA samples. We develop a three‐stage approach to infer the geography of fine population structure and draw conclusions with regard to the strategic role of geography in the wider inquiry into bio‐social relations.

## Data

### The sample

We drew on genotyped DNA‐sequences of 2039 volunteers, which were collected in 2008/09 as part of the Wellcome Trust‐funded study of ‘The People of the British Isles’ (POBI). The sample was designed with a view to representing known population subgroups as well as to identify fine population structure within the broader British population (Leslie *et al*. 2015; Winney *et al*. 2012). For this purpose, volunteers were only recruited to the study if their four grandparents were born in rural Britain and no more than 80 kilometres apart from each other. ‘Rural’ areas are held to be more homogeneous and were defined as lying at least two kilometres away from towns with present‐day populations of 125 000 inhabitants or more (see Winney *et al*. 2012). Since the four grandparents account for the entire genetic material of an individual, proximate birthplaces of grandparents reduce the likelihood of admixture, especially if they were born at a time when rural migration destinations were rare. The mean birth year of grandparents was 1885.

**Figure 1:**
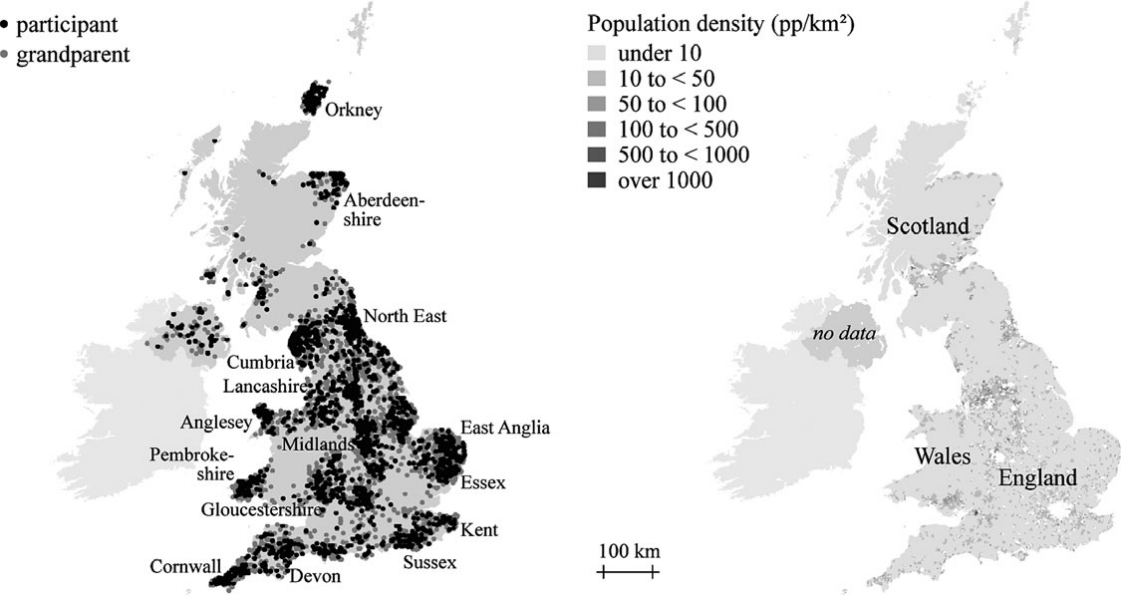
The ‘POBI’ sample locations (left) and population density of ‘rural’ parishes in 1881 (right)

Figure 1 (left) shows the geocoded locations of each volunteer's four grandparents for whom the locations were available (n = 2019) alongside the ‘ancestral mean geographic location’ of each volunteer, defined as the centroid of the four points. The geographic distribution of the sample is uneven, as we deem typical for DNA scans taken from participants based on their consent and the requirement of volunteers to attend events or clinics. In the case of the POBI sample, the restriction to recruit only from rural areas additionally skewed the geographical distribution. While the sample covers large parts of northern, central and southern England, the area of mainland Scotland is underrepresented. By contrast, other areas are overrepresented, consistent with the POBI design: Anglesey and Pembrokeshire in Wales, Orkney and Aberdeenshire in Scotland and Cumbria, East Anglia and Gloucestershire in England.

The motivation for restricting sampling to rural families that are long established in their respective areas was to emphasise historic spatial patterning by excluding genetic material that had been recently imported into localities – for example, through rural–urban or international migration. The sample thus presents a historic pattern of the genetic traits of the grandparents’ generation that have been passed down to the present‐day established residents. In view of historic rates of mobility and migration, which chiefly occurred over short distances (Pooley and Turnbull 1998, 65), it may be speculated that the spatial genetic structure has remained relatively stable since historic invasions prior to the 12th century and the wide advent of the system of given and family names.

### Population register

In order to provide a population‐wide basis to comparison, an appropriate population register should satisfy the following criteria:

1. it should contain surnames as proxies of biological characteristics;
2. it should be a nearly complete micro‐dataset referring to the same time period that the sample material refers to, i.e. to the time of the grandparents’ births (around 1885);
3. it should allow removal of surnames known to have been imported from abroad in the more recent past and which may be considered to be ‘native’ as a consequence;
4. it should map to the spatial extent of the sample with similar inclusion/exclusion criteria, and more specifically it should only contain rural records according to the definition of ‘rural’ used in the sample design.

Population registers that hold surnames (criterion 1) and at the same time provide a nearly complete set of micro‐data for the time period of interest (criterion 2) are the digital micro datasets of the 1881 Censuses of England, Wales and Scotland (Schurer and Woollard 2000a, 2000b). These datasets are available for academic use through the ESRC‐funded UK Data Service (www.ukdataservice.ac.uk). There are no 1881 Census data available for Northern Ireland. Records that refer to populations that likely have a recent migration history were removed using a surname classification tool (Mateos 2007): only records of people with Anglo‐Saxon, Irish, Scottish, Cornish and Celtic names are retained (criterion 3). The 1881 Census of Population holds information about the residential parish of each record, which are partially geocoded (Southall 2012, 2014) and can therefore be linked to the geographic definition of rural areas used in the POBI project (i.e. areas that are at least two kilometres from towns with present‐day populations of 125 000: criterion 4).^1^ Applying each of the four criteria results in a population that can be considered to best match a potential reference population for the sample (Table 1).

**Table 1:** The reference population after application of the four inclusion criteria

	Population^a^	Surnames	Parishes^b^
Total	29 912 298	518 153	7203
With local surname	27 213 993	61 286	7203
With local surname and rural	18 692 871	57 511	6848

^a^Population registers are not complete. ^b^Some parishes have been grouped to meet a minimum population threshold of 750 people.

## Analytical design to create a regionalisation based on population structure

The general strategy to regionalise Great Britain based on population genetic structure and surname geographies comprises three stages, in which each POBI participant's genetic profile is compared to the 1881 surname ‘profile’ of the sampled individual's grandparents’ residential area. Genetic profiles can be generated by measuring the genetic similarity between each pair of participants. This similarity is termed ‘co‐ancestry’ because it can be interpreted as the probability of any two participants being descended from a common ancestor. Participants can be classified into any number of *l* groups based on co‐ancestry, and here we will describe these groups as ‘genetic clusters’.

An equivalent procedure can be followed for areas based on their surname profiles. The similarity of surname profiles between areas, which is termed ‘isonymy’, can be used to classify areas into any number of *k* groups. Combining these approaches, we can link the genetic cluster of a POBI sampled individual with the isonymy group based on the residence of the participant's grandparents. The following three‐stage approach is then carried out.

1. Global correspondence. We establish the agreement between any combination of *l* genetic clusters and *k* isonymy groups. For instance, we may group participants into two genetic clusters and two isonymy groups and determine the proportion of participants that belong to the same pairing of genetic cluster and isonomy group. By extension, this can be performed for any partitioning, e.g. two genetic clusters and three isonymy groups, three genetic clusters and three isonymy groups and so forth. Using an index of partition agreement, we identify the combinations that best match based on how many participants of the same genetic cluster fall into the same isonymy group.
2. Local correspondence. Having identified the best‐matching combinations of genetic clusters and isonymy groups, we proceed to measure the degree to which each isonymy group is likely to reflect ‘true’ population genetic structure based on evidence from the POBI sample. We use two indices, Dominance and Distinctiveness. Dominance measures how homogeneous each isonymy group is in terms of the number and size of genetic clusters it contains spatially; and the Distinctiveness of each isonymy group measures the extent to which the genetic clusters it contain occur in other isonymy groups. We combine these two indices to form an overall index of Regional Integrity.
3. Regionalisation. We identify the constellation of isonymy groups that maximises Regional Integrity. We start with the most disaggregate, best‐agreeing combination identified in the measurement of global correspondence (Stage 1) and merge the isonymy groups that show a low value of Regional Integrity. Iteratively, we arrive at the optimal regionalisation of Great Britain, which best spatially represents population structure based on the evidence found in the genetic sample and the population register.

*R* (R Development Core Team 2014) was used as the software with the *base*,* classInt*,* reshape2* and *plyr* packages for data management (Bivand 2013; Wickham 2007, 2011), *stats*,* clue* and *modeest* for statistical analysis (Hornik 2005, 2015; Poncet 2012) and *maptools*,* rgeos*,* ggplot2*,* sm* and *spatstat* for geospatial analysis and visualisations (Baddeley and Turner 2005; Baddeley *et al*. 2013; Bivand and Lewin‐Koh 2014; Bivand and Rundel 2014; Bowman and Azzalini 2014; Wickham 2009).

The following sub‐sections provide more technical details on how genetic profiles and isonymy groups are defined and how the indices of partition agreement and Regional Integrity are produced. The less technically interested reader may skip these sub‐sections and continue with the results.

### Preparation: linking sample and register

DNA scans of many individuals can be summarised in a similarity matrix measuring the percentage of DNA that most likely stems from a near common ancestor between each pair of individuals in the sample (for more details see Lawson *et al*. 2012; Leslie *et al*. 2015). This ‘co‐ancestry matrix’ can be used for genetic profiling (often involving cluster analysis of individuals producing a classification of DNA profiles). Lawson *et al*. (2012) produced a co‐ancestry matrix for the POBI sample and Leslie *et al*. (2015) completed the genetic profiling with an additional, probabilistic adjustment to the resulting assignments of individuals to 53 genetic profile classes (see Leslie *et al*. 2015, supplementary material p. 2, for details). Both the co‐ancestry matrix and the adjusted profile assignments were provided for geographical analysis.

A comparable procedure can be applied to surnames for small areas. We calculate local concentrations of surnames in 1881 parishes and compare each pair of spatial units with respect to similarity of their surname compositions. This logic follows Lasker's (1977) application of isonymy – ‘the recurrence of the same surnames in different ancestral lines in the same pedigree’ (Lasker 1969, 309) – to estimate relatedness between or genetic similarity of populations based on the relative frequency of common surnames:

(1) $$\eta_{ij}=\sum_{s}\frac{n_{s.i}n_{s.\;j}}{2n_{i}n_{j}}$$

where *n*_*s.i*_ and *n*_*s.j*_ are the number of bearers of surname s in populations i and j respectively.

When the logic of isonymy is transferred to areas, the repeated calculation for each area results in a matrix of isonymy between each pair of areas. This isonymy matrix is comparable to the co‐ancestry matrix; the former describes similarity between areas in terms of isonymy, while the latter describes similarity between individuals in terms of hypothetical co‐ancestry.

### Stage 1: Measuring global correspondence

We compare the geographical distribution of genetic profiles with the geography of different surname mixes in local areas (parishes). The surname mixes are calculated by inverting the isonymy matrix to a distance matrix (using the negative logarithm of the matrix values) and running Ward's hierarchical clustering algorithm on it. The result is a taxonomy of areas by their surname compositions, available for any number of isonymy groups *k*. A similarity index, the Adjusted Rand (Hubert and Arabie 1985), is used to measure the correspondence between two alternative partitions of the POBI sample: one partitioned by genetic cluster, and the second by isonymy group of the local area.

The generic formula of the Adjusted Rand is

$$R_{adj}=\frac{R-E(R)}{\max(R)-E(R)}$$

where

(2) $$R=\left(\begin{array}{c} n_{cons}\\ 2 \end{array}\right){\left(\begin{array}{c} n\\ 2 \end{array}\right)}^{-1}$$

where *R* is the actual index value, *E(R)* the expected value, *max(R)* the maximum possible value, *n* all observations and *n*_*cons*_ observations in consonant pairs.

For more details on the reproduction of the statistic see Hubert and Arabie (1985) and Albatineh *et al*. (2006). The index ranges from −1 to +1, where +1 indicates identical partitioning by the two partitions *k* and *l* and −1 opposing partitioning. A value of 0 indicates random partitioning or no correspondence.

### Stage 2: Developing local indices of Regional Integrity

In a second step, we seek to establish the local correspondence between co‐ancestry and isonymy. For each isonymy group, we measure the degree of correspondence to the co‐ancestry information of the sub‐sample that falls into the spatial extent of the isonymy group. Comparable in kind to studies of alpha and beta diversity in ecology (Magurran 2004), we break down local correspondence into Distinctiveness and Dominance.

We measure Distinctiveness as the extent to which the genetic clusters found in areas of the same isonymy group do not also occur in other isonymy groups. If, for example, 100 per cent of observations of a genetic cluster occur only in one isonymy group, the genetic cluster is characteristic of that isonymy group and thus contributes to its degree of Distinctiveness. The logic is similar to that of the Location Quotient (LQ), which is a quantity that is widely applied in geography to describe relative density of phenomena in one region relative to other regions (Burt *et al*. 2009). We modify the LQ to range between 0 and 1 as follows:

(3) $$DIS_{k}=\sum_{l}\frac{n_{kl}^{2}}{n_{k}\cdot n_{l}}$$

where *n*_*kl*_ is the number of observations in isonymy region *k* that belong to genotype *l*.

Hence for each isonymy group, Distinctiveness is the sum of the regional share of total observations that belong to a given genetic cluster weighted by the prevalence of the genetic cluster within the group. In other words, the Distinctiveness can be conceived of as weighted proportional LQ of all genetic clusters that occur in one region. Since it uses proportions, the Distinctiveness index can be interpreted as a probability that the regional population is distinct from the remaining regional populations.

The second component, internal homogeneity, can be expressed as the Simpson Index of Dominance (Simpson 1949). The index measures the degree to which one group is dominant relative to the remaining groups.

(4) $$DOM_{k}=\sum_{l}{\left(\frac{n_{kl}}{n_{k}}\right)}^{2}$$

It describes the probability that two randomly chosen individuals from a region belong to the same genetic cluster.^2^ Yet, while a probabilistic reading is useful, it should be noted that some values of the indices are more likely than others because of unequal sizes of isonymy groups. It is necessary to adjust for chance, as is done with the Rand index (Equation (2)), whereby we subtract the expected index value from the actual value and divide it by the difference between the maximum possible value and the expected value. The maximum possible value for both Distinctiveness and Dominance is 1 in both cases. The expected value for Distinctiveness of a group, however, is equal its share of observations; hence the unadjusted index is not comparable across regions. Similarly, if genetic clusters were distributed randomly, the expected Dominance index in each region would be 1/*L* where *L* is the number of genetic clusters. Under adjustment, a value of 0 indicates randomness and 1 perfect departure from randomness.^3^

A combined index of Regional Integrity can be calculated by multiplying both indices together.

(5) $$RI_{k}=DIS_{k.adj}\cdot DOM_{k.adj}$$

where the subscript *adj* indicates the adjusted version of the index.

### Stage 3: Identifying the optimal regionalisation

Based on the Adjusted Rand Index and sample size, we identify the combination with the highest level of granularity that seems acceptable. We then proceed with the following steps.

1. We merge the regions to the next coarsest level as indicated by the next local maximum of the Adjusted Rand index.
2. If the Regional Integrity index of the merged regions is higher than all its sub‐regions, the merger is accepted; otherwise we keep the sub‐region with higher Regional Integrity and merge the remaining sub‐regions.
3. We recalculate local correspondence indices for the resulting partition.
4. We repeat steps 1 to 3 on the resulting partition until no further improvements occur or the coarsest level of regionalisation is reached.

## Results

### Global correspondence: which level of regionalisation seems best?

**Figure 2:**
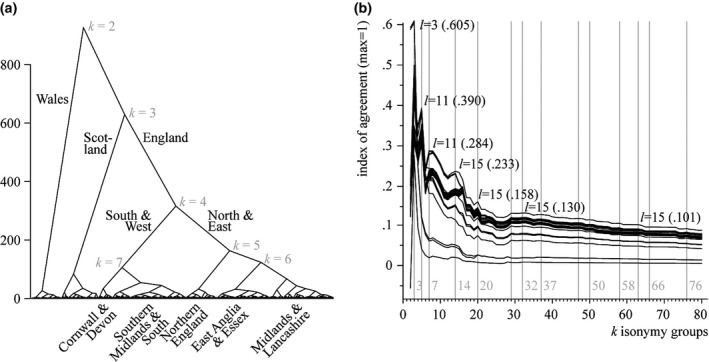
Dendrogram of 1881 rural parish groups of Great Britain classified by isonymy (left) and Adjusted Rand Index values of agreement between *l* genetic clusters and *k* isonymy groups (right). Each line represents a genetic cluster solution with *l* ϵ {2..53}

As described above, we cluster the inverted isonymy matrix using Ward's clustering algorithm of parish groups for 1881 with population weights to generate a full range of between 2 and 80 clusters of areas with distinct surname compositions. The dendrogram is a useful way to display the entire taxonomy of areas, showing the distances (in statistical space) at which individual areas (at the bottom) are assigned to iteratively increasing clusters (Figure 2a). Examining the resulting taxonomy of areas for their geographical attributes, we can note that a strong regional grouping of areas by isonymy emerges from this classification procedure, although no geographic information was processed as part of it. Parishes successively merge into regional agglomerations, such as Wales, northern and southern Scotland, Cornwall, Devon, the Midlands, southern England, northern England and so forth. These clusters are further merged to larger regional agglomerations that form Wales, Scotland and southern and western, northern and eastern England. The latter merge into England, which then together with Scotland and finally Wales make up a single cluster of all areas. This taxonomy is consistent with previous findings (Cheshire and Longley 2012; Longley *et al*. 2011), although it should be noted that urban parishes are excluded here, as is the whole of Northern Ireland, for which no 1881 Census data are available.

Based on a similar clustering procedure, study volunteers have been assigned a genetic cluster (a group of volunteers whose genetic characteristics indicate that they likely share similar ancestry) at successive stages. At each level, participants have been grouped into 2, 3, 4, etc. up to 53 clusters resulting in an assignment matrix, developed and provided by Leslie *et al*. (2015). Together with the isonymy class of their ancestral places of birth, the genetic cluster and area isonymy class of each participant can be compared. First, we assign each volunteer his or her genetic profile for each of the *l* cluster solutions (*l* ϵ {2..53}). We then append to the volunteer information the membership of each isonymy group *k* (with *k* ϵ {2..80}) based on the location of the grandparents’ birth places. Since there are four grandparents who may each reside in a different isonymy group, the most common group among the four grandparents is assigned to the participant. There can be ties in relatively rare circumstances (7.5 per cent for *k = *20 isonymy groups) when two grandparents or each single one belonged in a different isonymy group. In this case, we selected one of the groups randomly.

We then measure the level of agreement between various *l* and *k* using the Adjusted Rand similarity index (Hubert and Arabie 1985), which calculates a ratio between consonant pairs and dissonant pairs of observations and adjusts these for chance. If, for example, two participants are both assigned to an isonymy group *X* and a genotype *Y*, they are consonant pairs of observations. If another participant is assigned to *X* and genotype *Z*, she forms a dissonant observation to the other two. The index ranges from −1 to +1, where 0 indicates random partitioning or no correspondence, 1 identical partitioning and −1 opposing partitioning, which is the case when the entire sample is grouped into one class according to one partition and each sample record is in a distinct class in the other partition. Note that the index is aspatial; it measures the level of agreement between two classifications irrespective of geographical proximity or contiguity.

Plotting the index value of each partition (or cluster solution) of genetic profiles *l* against each of isonymy group *k* presents a picture of declining correspondence as the number of classes *k* increases. We are interested in the combinations of *k* and *l* where the index exhibits pronounced local peaks in order to identify those partitions that agree most. The index shows a global peak at *l *=* *3 genetic clusters and *k *=* *3 isonymy groups with an index of .605 (Figure 2b). The next best local peak is at *l *=* *11 genetic profiles and *k *=* *5 isonymy groups, followed by other local peaks at *l *=* *11 and *k *=* *7, *l *=* *15 and *k *=* *14, *l *=* *15 and *k *=* *20 and so forth. At *k *=* *13 isonymy groups, the partition with *l *=* *15 genetic profiles remains superior to any other genetic profile partition.

**Figure 3:**
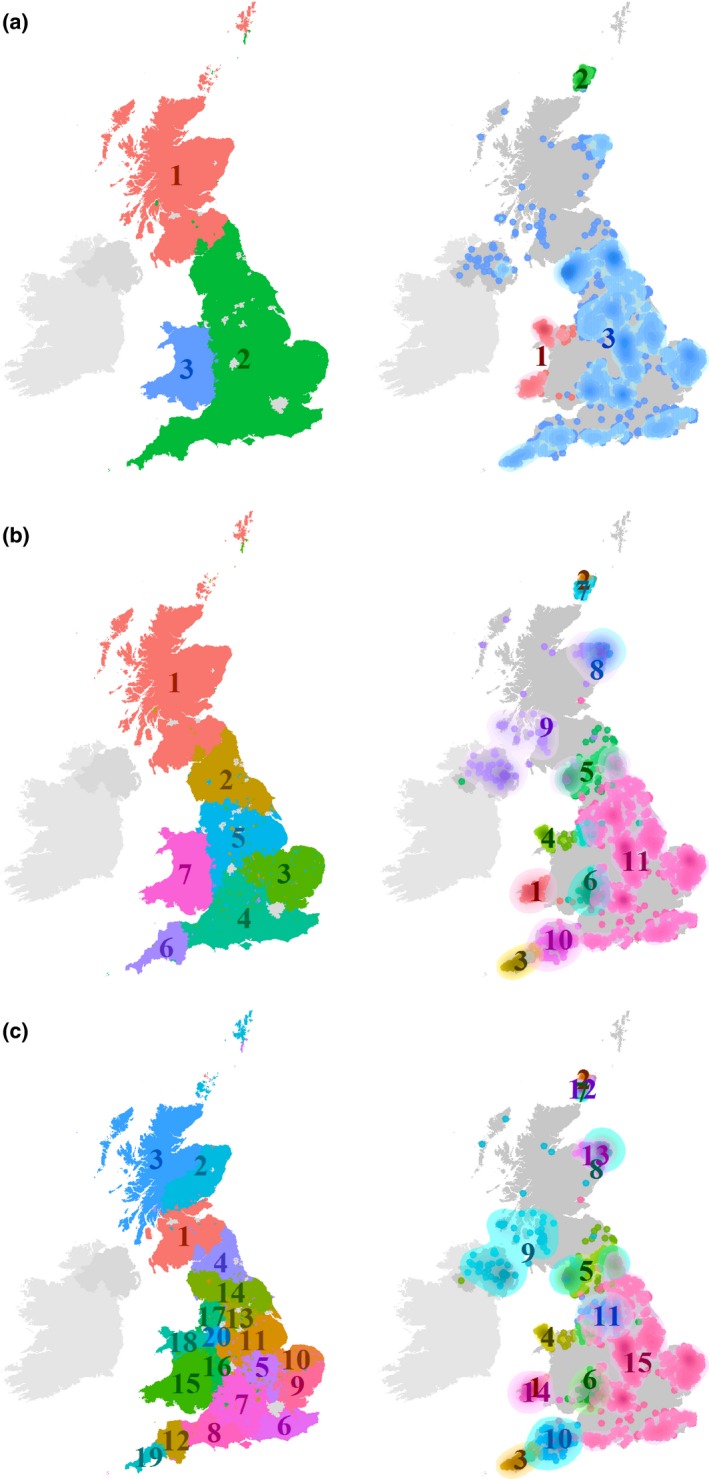
Isonymy groups (left) and the geographic distribution of genetic clusters (right). (a) *k* = 3 isonymy groups and *l* = 3 genetic clusters with *R*_*adj*_ = .605. (b) *k* = 7 isonymy groups and *l* = 11 genetic clusters with *R*_*adj*_ = .284. (c) *k* = 20 isonymy groups and *l* = 15 genetic clusters with *R*_*adj*_ = .158

Figure 3 shows the geography of these selected combinations of isonymy groups and genetic clusters. The left‐hand maps show parishes for 1881 coloured by isonymy group. The right‐hand maps show the POBI sample using the centroid location for each set of four grandparents’ locations, coloured by genetic cluster. We use kernel density estimation with varying bandwidth as a visual aid to highlight geographical concentrations of genetic profiles, where they exist. The bandwidth was chosen to be slightly wider than the 95th percentile of the distribution of nearest neighbour distances among observations of each group. This has proven to usefully display tendencies of spatial concentration as it captures the majority of point‐to‐point distances within a group.

The partition with *k *=* *3 isonymy groups divides Great Britain into three regions: Wales, England and Scotland (Figure 3a). In the genetic cluster solution with *l *=* *3, three genetic clusters can be identified that broadly correspond to Wales, England‐Scotland and Orkney.^4^ With the Adjusted Rand index (*R*_*adj*_) of 0.61, this combination indicates high certainty about the presence of population structure between those regions.

The next partition with a peaking *R*_*adj*_ of .284 comprises a combination with *k *=* *7 surname regions and *l *=* *11 genetic clusters (Figure 3b). One of the isonymy groups covers Scotland almost entirely. Four genetic clusters concentrate in this region, with two distinct genetic clusters detected in Orkney. A second isonymy group emerges in northern England, where a number of spatially overlapping genetic clusters can be observed. It appears that in isonymy groups 1 and 2, the genetic diversity of populations is higher than the mix of surnames in Scotland and northern England would suggest at this level. Isonymy groups 3, 4 and 5 encompass the widespread genetic cluster 15, which reaches into the northern English isonymy group 2. A sixth isonymy group emerges in south‐west England, encompassing two concentrations of genetic clusters there: one in Cornwall (genetic cluster 3) and one in Devon (genetic cluster 10). Isonymy group 7 – a Welsh cluster – corresponds to the concentration of three distinctive genetic clusters.

Figure 3c provides a more fine‐grained picture of isonymy and co‐ancestry, albeit with lower correspondence, as indicated by an *R*_*adj*_ of .158. The seven isonymy groups split into further sub‐regions. Scotland now consists of three sub‐regions and Wales splits into a southern (15) and a northern (18) cluster with two additional isonymy groups (16, 20) along the border between England and Wales. These four clusters in and around Wales correspond geographically to genetic clusters 1 and 14, 4 and 6. The south‐west of England now splits into two distinct isonymy groups covering Cornwall (19) and Devon (12) and thereby corresponding to genetic clusters 3 and 10. The isonymy groups in central and southern England suggest much greater population heterogeneity than is indicated by co‐ancestry: here the genetic cluster 15 prevails. The opposite can be observed in Orkney: here we find more genetic heterogeneity than surnames would suggest.

### Local correspondence: geographical variation in correspondence

The succession of isonymy maps suggests different levels of regionalisation that best reflect the population in genetic terms. Yet, a regionalisation purely based on global correspondence of genes and surnames conceals local variations in the heterogeneity of each measure: it is clear from Figure 3 that the genetic heterogeneity found in the sample varies geographically, and the same is also true of heterogeneity in naming conventions in different parts of the country.

Based on this reasoning, we define high local correspondence as a property of an isonymy group when it encompasses observations that, in terms of their co‐ancestry, are distinct from other isonymy groups (*Distinctiveness*) and simultaneously similar to each other (*Dominance*). Together the two components convey a sense of confidence we can ascribe to the degree to which an isonymy group truly represents population structure. We call this the *Regional Integrity* of an isonymy group in terms of the population structure found in the sample.

**Figure 4:**
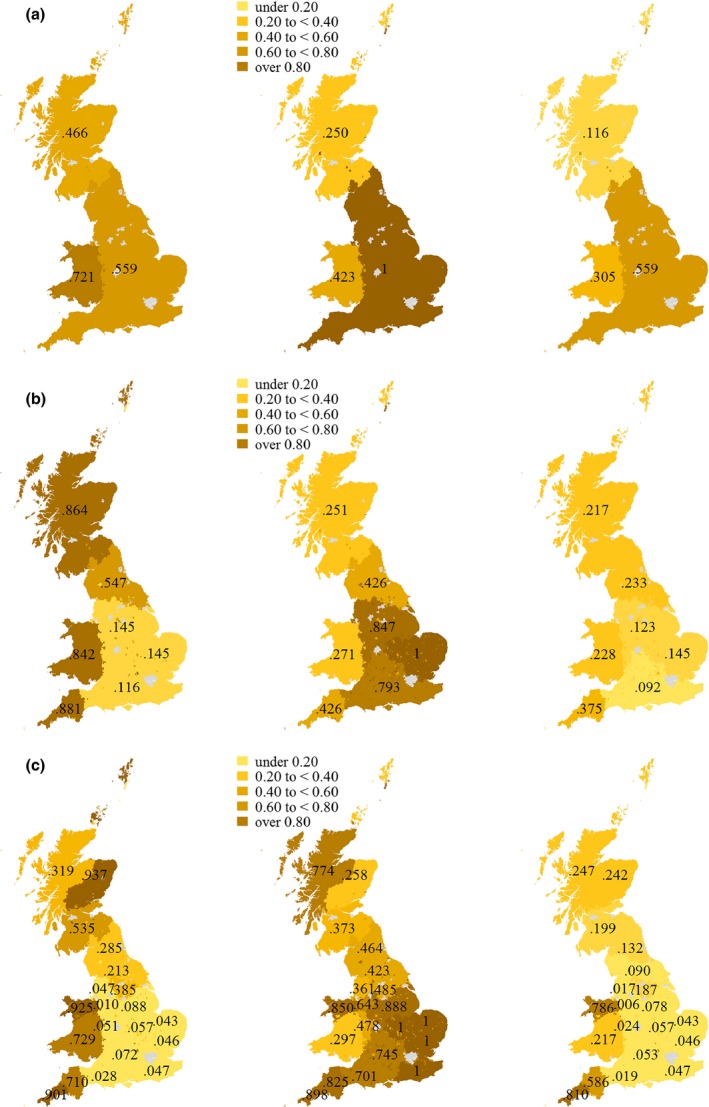
The distinctiveness (left), dominance (middle) and regional integrity (right) indices for each combination with high global correspondence. (a) *k* = 3 isonymy groups and *l* = 3 genetic clusters. (b) *k* = 7 isonymy groups and *l* = 11 genetic clusters. (c) *k* = 20 isonymy groups and *l* = 15 genetic clusters

Figure 4 shows Distinctiveness, Dominance and Regional Integrity for all three most agreeing combinations of isonymy regions and genetic clusters. For the first combination (Figure 4a), the one with the highest level of global correspondence, the Welsh isonymy region has the highest level of Distinctiveness with a value of .721. The English isonymy region has the second highest level of Distinctiveness (.559). Although it encompasses 89 per cent of genetic cluster number 3 (cf. Figure 3a) and therefore leads us to expect high Distinctiveness, its larger size compared to other regions – 80 per cent of observations fall in this region – produces a higher expected value of Distinctiveness and hence depresses the index value. The Scottish isonymy region exhibits the lowest level of Distinctiveness (.466), which can be explained by the fact that, although Scotland hosts the distinctive population that is classified as genetic cluster 2, these comprise just 40 per cent of the observations in Scotland. The remaining 60 per cent belong to the mostly English genetic cluster 3, which reduces Distinctiveness and even more so Dominance. In terms of Dominance, the English region is the top‐scoring one because within its regional extent, only genetic cluster 3 prevails. The Welsh region hosts all observations with genetic cluster number 1, but the observations in the England–Wales border region, which make up a quarter, belong to genetic cluster number 3. These patterns attest to a higher level of Regional Integrity to the English region, followed by Wales and Scotland. Given that in the latter two regions, the Distinctiveness is higher than Dominance, a more granular division may lead to improvements in Regional Integrity. Conversely, it is less likely that Regional Integrity in the English regions can be improved unless further splits in terms of isonymy and granularity are spatially congruent.

The next combination with seven isonymy regions and eleven genetic clusters changes some of the patterns (Figure 4b). Now, the Scottish region exhibits a higher level of Distinctiveness than other regions. This is because the genetic clusters that formerly extended across southern Scotland and England now split into more local genetic clusters number 5, 8 and 9 (cf. Figure 3b). The latter two are largely confined to southern Scotland and only a minority of the genetic clusters 5 and 11 remain in this region. Given a resulting lower expected index value of Dominance, the more granular genetic clusters increase homogeneity, adding up to an overall improvement of Regional Integrity for the Scottish isonymy region. The unchanged Welsh region has greater Distinctiveness following regional subdivision of the formerly English genetic cluster into new genetic cluster number 6, which prevails in the England–Wales border region. Given further splits, population heterogeneity increases and therefore the Dominance score remains low in comparison with other regions. A highly distinctive region has emerged in the south‐west, covering Cornwall and Devon. Given that this new region corresponds to two highly localised genetic clusters, the level of Dominance is lower. The picture reverses for England. While most English regions lose Distinctiveness, their internal homogeneity remains high as the widespread genetic cluster 11 is dominant among their corresponding observations in the sample. The resulting map of Regional Integrity appears rather invariant. The most consistent region is the Devon and Cornwall cluster, while the least consistent regions are the south‐eastern isonymy groups. Regional integrity has improved for the Scottish region compared to the coarser partitioning, while it has decreased for Wales. There and in Devon and Cornwall, Dominance is lower than Distinctiveness which indicates that greater integrity could be gained by further regional division.

The final combination with the highest granularity confirms the tendencies found in the previous one (Figure 4c). Twenty isonymy clusters and 15 genetic clusters reduce the number of highly distinctive regions to one in north‐eastern Scotland (which includes parts of the Orkney and Shetland islands), two Welsh regions, Cornwall, Devon and to a lesser degree southern Scotland. The southern and eastern English regions are the least distinctive, which arises because the widespread English genetic cluster number 15 did not split significantly compared to the last combination. The latter is reflected in a high level of Dominance among the south‐eastern English isonymy clusters. Four of these show a value of 1, implying that there is only one genetic cluster present among the corresponding observations. The absolute number of observations varies between 89 and 155 in these regions. Now, the south‐western isonymy clusters exhibit higher values of homogeneity along with clusters in northern Wales and northern Scotland. At this higher granularity, it seems that the isonymy clusters reflect more homogenous populations in most parts of the country based on the sample information on co‐ancestry. Yet, Regional Integrity suggests that the lack of Distinctiveness in some of the isonymy clusters outweighs the benefits of viewing homogeneous populations. Merging some of the English regions, for example, would better reflect population structure in Great Britain than the highly granular solution. The only regions that show a high likelihood of Regional Integrity are Devon and Cornwall, and northern Wales. There is a second tier of intermediate values, which can be found in Scotland and southern Wales. This arises because of higher Distinctiveness in southern Wales and Scotland–Orkney, suggesting that another split of this region may improve integrity values.

### A triangulated geography of population structure in Great Britain

We use Regional Integrity values at various levels of granularity to derive a synthesised picture of Great Britain's geography of population structure, assembling the isonymy groups and genetic clusters that result in the highest Regional Integrity. This is done by an iterative process that begins with the most disaggregate combination of genetic clusters and isonymy groups, which is above a given threshold of global correspondence as measured by the Adjusted Rand Index. This threshold may be defined based on sample size, but essentially it is a subjective decision as to which level of the Adjusted Rand Index is deemed acceptable. The regions are then merged to the next coarsest level, and, if Regional Integrity of the merged region improves compared to all previous sub‐regions, the merger is accepted; otherwise, only the sub‐region with the higher Regional Integrity than the merged region is retained and all other sub‐regions are merged. In this way, at each iteration, the improvement of Regional Integrity is measured and the process of aggregation terminates when no further improvements occur or the coarsest level of regionalisation is reached.

**Figure 5:**
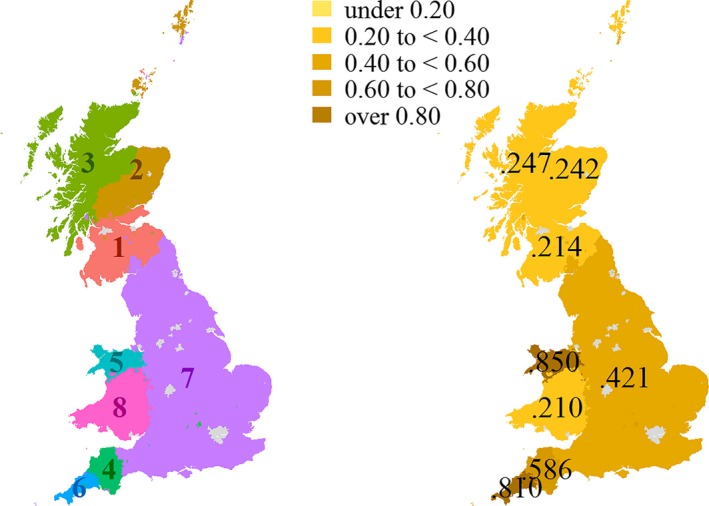
The synthesised regions of population structure in Great Britain (left) and their corresponding regional integrities (right)

Figure 5 shows the resulting synthesised geography of population structure in Great Britain. Note that mergers of sub‐regions that belong to different parent regions are not allowed. The resulting partition corresponds to eight isonymy regions based on the 15 co‐ancestry groups. Scotland is now composed of far northern, north‐eastern and southern sub‐regions. The large English region comprises all former sub‐regions except Devon and Cornwall, which each remain separate. Wales is divided into a northern and southern part. The resulting Regional Integrity indices range between .210 in southern Wales and .850 in Cornwall.

As a whole, the map highlights those areas of Great Britain where we can be most confident about the existence of population structure – northern Wales and Cornwall – given the information in and geographic distribution of the POBI sample viewed jointly with the geographic concentration of surnames. We should be more cautious in Scotland and southern Wales, where further sampling would be necessary to increase certainty about population structure. By viewing the components of Regional Integrity, we find that the high uncertainty in southern Wales arises because of internal heterogeneity, that is lack of Dominance among the prevalent genetic clusters. Further sampling in this region may help to attain more clarity about population structure in southern Wales. This may well lead to a further split of southern Wales or a merger with adjacent regions. The same conclusion can be drawn for southern Scotland, while the contrary applies to northern Scotland, where the sample suggests a high level of homogeneity but a lower level of Distinctiveness. In Scotland, the sampling is particularly uneven, with nearly half of all Scottish observations made in Orkney. Again, a more geographically representative sample may increase confidence about population structure in Scotland and improve the integrity of regional partitioning.

In summary, by means of the algorithm that maximises Regional Integrity, we are able to develop the most robust regionalisation of population structure in Great Britain given the joint evidence of a genetic sample and a corresponding nearly complete population register. Geographical heuristics facilitate a quantitative assessment of uncertainty and highlight regions where more information is needed, be it through further in‐depth study of local surname patterns or measuring genetic characteristics in the corresponding sub‐sample.

## Discussion: implications for geography and bio‐social research

This paper deploys geographical heuristics to contextualise a present‐day study of the human genetics of Great Britain using historic Census of Population data. The heuristics match present‐day genetic data with historic surname geographies and then calculate a number of measures of correspondence. Global correspondence measures provide a general picture of regional differences in population structure at different levels of granularity, confirming pronounced differences between three of the UK countries as well as regional differences between northern England, south‐western England, and northern and southern Wales. Finer scale regionalisations are less distinctive but are nevertheless important in some parts of the country, such as Devon and Cornwall. After using global correspondence to filter the most matching partitions of isonymy and ancestry data, partly bespoke measures of local correspondence are used to determine where isonymy and co‐ancestry correlate. This information improves the regionalisation and addresses vagaries that are a consequence of the highly selective genetic sample, the genotyping and overlapping geographies of genealogy.

### Significance and interpretation of the regionalisation

The resulting regionalisation draws the most likely boundaries of fine population structure based on the joint evidence emanating from genome and surname geographies. Despite the apparent crispness of the regionalisation, the boundaries should not be understood as representing sharp transitions of bio‐genetic characteristics but rather mark a possible classification based on gradients of bio‐genetic similarity. The Regional Integrity index itself reflects the degree to which regional populations may be considered to be distinctive. The significance of difference is also an outcome of the level of granularity of both classification by co‐ancestry and classification by isonymy: the more intricate the classification, the less marked are the differences between them in absolute terms.

In view of the relevance of population structure, the regionalisation provides important information for bio‐social research. Bio‐social relations are likely to take on different patterns in each of the different regions, if the regions effectively represent underlying population structure. Although the regionalisation is generated from data that indeed pertain to an ancestral generation, the regional patterns have enduring resonance today, as genetic material is passed on over generations, thus carrying forward population structure of ancestral generations. Even the cumulative effects of more recent migration are unlikely to mask the genetic divergence between sub‐populations (or random genetic drift: Hartl and Clark 1989, 306), and our regionalisation is of relevance beyond the long‐settled rural populations of Great Britain. If, in addition, we consider that the vast majority of people remain within 100 kilometres of their birthplace (Pooley and Turnbull 1998, 65), the present‐day population structure of most of the country is unlikely to have profoundly changed.

The Regional Integrity index additionally provides a means to assess how well population structure might be represented if, for example, urban areas were included in the reference population or a contemporary population register were used in the comparative analysis. In both cases, global and local correspondence measures, notably the Regional Integrity index, would decrease in some regions. More generally, our approach could be used to measure the agreement of a given genetic sample with a range of different reference populations in order to ascertain the most appropriate population frames of the sample. In this way, the uncertainty resulting from the typically highly skewed and selective nature of genetic samples can be addressed.

Our regionalisation may also be a useful resource to inform sample designs of future genetic studies. In the context of medical and health geography, for example, sample designs that recruit participants or selects sufficient observations from each of the eight regions may be better suited for generalisations about the entire British population than samples designed only to reflect conventional demographic and social population characteristics. Studies that seek to investigate particular associations may benefit from sampling only from one region where the population is likely to be more homogeneous in terms of genetic characteristics. Where there are databases that depend on consent (such as the UK Biobank), separate analyses for each of the regions or other ways of accounting for the regional manifestation of population structure may be useful.

The regionalisation of population structure also identifies the parts of Great Britain where we should be relatively more wary of generalising about bio‐social or biological phenomena. Where isonymy and co‐ancestry align, we can be more confident in attributing biological phenomena (e.g. phenotypes) to the regional sub‐population in general, and bearers of regionally concentrated names (and their relatives) in particular. Where they do not align, we have less basis to ascertain the degree to which a phenomenon is present in the sub‐population. This applies, for instance, to Scotland and southern Wales, where we do not know whether the genetic clusters found are characteristics of particular, sampled individuals, sampling sites, a locality or the wider region. This uncertainty limits our ability to assess the importance of phenomena for the population at large.

Whichever the strategy, triangulation by using ancillary data is necessary to establish a valid basis for inference and generalisations. Genetic samples are likely to remain constrained by prior assumptions on the genetic structure of populations, arising out of the results of previous research. They are thus likely to be highly selective and geographically skewed and may also lack a clearly defined source population in time and space. Our findings suggest that this selectiveness can be countered to some extent by use of surnames as population‐wide genetic indicators, thus providing a more robust basis for inductive generalisations.

### Limitations

Some limitations of this approach should be noted. First, surname geographies are shaped by processes that may not accompany genetic diffusion. The consistency of surname adoption across space and time and the nature of naming conventions affect the signal quality of a surname with respect to population structure. In Wales, for example, where a relatively small number of surnames were adopted at a later period than in the rest of the country, surname geographies are much more invariant and thus less likely to pinpoint population structure. The wide use of diminutive surnames (e.g. Johnson, Williamson) and common occupation‐related metonyms (e.g. Smith, Carpenter) further obscures detail – diminutive surnames are very common right across Wales and only indicate specificity to this territory, while metonyms are often common across the entirety of Great Britain. Thus while our analysis is informative for most names, this is not the same as saying that it holds for a similar proportion of name bearers. Although grouping of spatially co‐occurring surnames should at least in part counter these difficulties, the impact of naming conventions and histories on surname–gene correlations at the area level requires further research. The scale of migration and mixing between migrants and longer settled residents will further weaken the ecological link between genes and surnames over time. Indeed, surnames only function as ancillary correlates to genetic population structure where surnames are hereditary, where naming conventions have been regionally variant at a sufficiently granular geographic scale and where the majority of the population does not migrate or migration origins and destinations are sufficiently definite that they can be accommodated. Existing studies on surname geographies suggest that most European and American countries, and Japan, satisfy these criteria (Cheshire 2014). While ancillary information is required to ascertain the wider population, a genetic sample is required, conversely, to measure the individual characteristics that are needed to align surname geographies with underlying population structure.

Second, the result of combining data on surname geographies and genes is in part a manifestation of the sample design of the POBI project. The degree to which a region is distinct and homogeneous is still affected by the number of observations that fall within a region and how many genetic clusters can be identified; both characteristics are in turn a result of sample design. It should be noted, however, that the Distinctiveness index penalises small numbers of observations to some extent. For instance, if the sample had been more skewed within England with the same observed genetic clusters, some English sub‐regions with more observations would have increased in their Distinctiveness. Conversely, regions with fewer observations would have lost Distinctiveness and thereby Regional Integrity. Since lower values result in mergers, and since more observations tend to produce higher values of Distinctiveness, regions with few observations tend to merge to larger entities, which is a desirable effect of the decision algorithm. In all of this, the sample size required to represent the parent population depends on the heterogeneity of the characteristic that is being measured: in the case of genes, this is unknown at the sample design stage, and in the case of surnames, this depends on geographies of naming conventions that at best bear only indirect and partial correspondence with genetic structure of the population. Bootstrapping may be a useful extension of the procedures set out here, in order to assign standard errors and confidence intervals to regional estimates. Yet, given sufficient observations in each isonymy group (at least 105), this was not felt necessary.

## Concluding remarks

Our view is that the analysis set out here combines the strengths of in‐depth genetic surveys with the pervasiveness of (historic and contemporary) population registers. In follow‐up genetic research it will be essential to devise sampling strategies that build on the results of the POBI project in the interests of improved generalisation. Given that bio‐social research straddles the purview of social similarity and biology, geography will have a crucial role to play in bridging the disciplines involved and offer solutions to inform research designs and interpretation of bio‐social phenomena, in particular as bio‐social research agendas become more developed and produce an increasing number of unstructured, highly selective samples that significantly depart from standards of social representativeness. In this sense, the POBI sample is not unlike many other population datasets. The three‐stage approach presented in this paper – measuring global correspondence, identifying local correspondence and bespoke regionalisation – may be an effective way to deal with such types of unstructured samples and support contextualised interpretations and valid generalisations that take into account the indefinite diversity of human populations.

## Notes

1 Digital boundaries for Scottish parishes of 1881 were not available at the time of the study. Therefore Scottish census records were geo‐coded using 1881 gazetteers (www.visionofbritain.org) matching 1951 parish boundaries with the parish name information available in the census file. The procedure resulted in approximate geo‐spatial delimitations of 1881 Scottish parishes. Professor Humphrey Southall and Dr Paula Aucott kindly made the 1881 gazetteer available for our study.

2 A geneticist may observe similarity to concepts related to estimating population structure from allele frequencies (Cavalli‐Sforza and Bodmer 1971, 343), for example by means of the Fixation Index *F*_*ST*_ (see also Hartl and Clark 1989, 282). Here, we seek to measure homogeneity within a regionally defined, hypothetical sub‐population and hence use an index commonly used in ecology.

3 In contrast to the Dominance index, the adjusted Distinctiveness index can fall below zero in extreme cases, for example when one region only encompasses one observation or indeed no observation at all. A negative Distinctiveness value would, however, indicate poor overall partitioning and would therefore be reflected in a very low adjusted Rand index.

4 Participants living in Northern Ireland are not processed in the analysis.
